# A De in the life of cholera

**Published:** 2011-02

**Authors:** Robert H. Hall

**Affiliations:** *National Institute of Allergy & Infectious Diseases, National Institutes of Health, Bethesda, MD, USA*

**Keywords:** Cholera toxin, De, El Tor, *Vibrio cholerae*

## Abstract

The 50-year commemoration of S.N. De’s seminal 1959 publication in *Nature* provides an opportunity to reflect on scientific discovery, recognition, and public health. De’s paper marked the first major conceptual advance in cholera research since 1884, when Robert Koch definitively identified *Der Kommabazillus* as the aetiological agent of cholera. Unfortunately, Koch reported that systemic toxinosis and multi-organ failure led to severe dehydrating diarrhoea, thereby mistaking cause for effect. As a consequence, while work on other microbial pathogens advanced into the development of vaccines and therapeutics, cholera research languished as scientists injected animals parenterally in decades of futile effort to develop an animal model of diarrhoea. This fundamental misconception in cholera pathogenesis was swept away when S.N. De used ligated loops of rabbit ileum to demonstrate lumenal fluid accumulation in the presence of *Vibrio cholerae* culture filtrates. After some delay, De’s observation of a diarrhoeagenic exotoxin became the founding principle of modern cholera research, vaccination, and treatment; and a burst of discovery saw *V. cholerae* transformed into the enteric pathogen best understood at the molecular level. The scientific basis for orally administering vaccines to induce mucosal immunity was established, and the success of oral rehydration, what has been described as one of the 20^th^ century’s most important medical advances, was explained.

Nobel laureate Joshua Lederberg wrote of De’s iconoclastic creativity, experimental skill, and observational mastery, and many other leaders in the field concurred. De was nominated for the Nobel Prize in Physiology or Medicine more than once. But despite the passage of half a century from De’s work, cholera remains a frustrating problem: we are clearly missing something. In reviewing the scientific and programmatic impact of S.N. De on cholera, it is clear that a defining victory against the disease is achievable, but only if basic scientific discoveries are relentlessly driven towards progress in public health.

## A brief biography of Prof. Sambhu Nath De

We heard a warm recitation of the early life of S. N. De in West Bengal from his friends and colleagues. The comprehensive biography by Sen and Sarkar^1^, showed that De was an exceptional student, rising from modest means to win a district scholarship to Hooghly Mohsin College. Again he did well, gaining scholarships and admission to Calcutta Medical College. De received his medical degree in 1939 and a Diploma in Tropical Medicine in 1942^1^.

## Training and research in the United Kingdom

Upon graduation, De began his research career as a demonstrator in pathology in Calcutta Medical College and later as a Ph.D. student of Professor Roy Cameron, Head of the Department of Morbid Anatomy, in the teaching hospital of University College, London. Cameron was a leading experimental pathologist, and University College and Hospital were premier research institutions as they are today. Roy Cameron was an Australian from a modest background who had also proved himself through rigorous academic work. He was unpretentious and not particularly impressed with the trappings of high society^2^. He was very familiar with India and the Commonwealth countries, and knew how to spot talent. Even though Cameron was one of the world’s leading pathologists, he was still hugely impressed by De, saying: “There is no doubt about it – he is one of the most outstanding of young men I have had through my hands and am prepared to believe that he is probably the best of the experimental pathologists in India… I am confirmed in my belief by other people’s opinion”^1^.

Cameron was the founding President of the Royal College of Pathologists, a fellow of the Royal Society, a recipient of the Royal Medal, and was knighted in 1957. According to Stoke and Drury^2^, Cameron was bemused when the flood of awards came. He remained adamant that good work did not require expensive apparatus or facilities, that data should be judged by their quality and not by the writing, and he zealously guarded his time for research and teaching.

De was a remarkable success in London. He persevered, published, and in 1949, after just two years, De completed the requirements for a research doctorate, graduated with a Ph.D. from the University of London, and returned to India. De again visited England in 1955 and 1962. The 1955 visit, supported by the Royal Society, was to present his work on enterotoxigenic *Escherichia coli* to the Pathological Society. The 1962 visit, supported by the Wellcome Foundation, was to receive his second research doctorate, a D.Sc. degree in Physiology from the University of London, but that was the last time De and Cameron were to meet. On hearing of Cameron’s death in 1966, De said: “My teacher the late Sir Roy Cameron was a source of constant inspiration to me and his encouragement kept up my spirits when low. His death put the last nail on my struggle against all these odds”^1^.

De could have easily built a career in the UK but chose to return home. Furthermore, De was becoming increasingly interested in experimental bacteriological pathology, what we now call microbial pathogenesis. And we all know the axiom: science must go to the disease. It is most unlikely that De would have studied cholera pathogenesis had he stayed. As Sen and Sarkar^1^ put it in their excellent biography: “De returned to Calcutta in 1949, and those of us who knew him from our student days were struck by the complete transformation in his outlook towards research, which from now on was to become his *yoga*.”

## Career in India

Before discussing De’s seminal work on cholera, it is certainly worth noting that over the years he published widely. He showed precise thinking, he described key problems, he gave simple explanations: he told stories of adventure into the natural world. He was curious about many things: rhinosporidiosis, synovial sarcoma, morphoea, hepatic necrosis with haemorrhagic fever, tuberculosis, paratyphi, and tetanus^1^. Then there came his brilliant work, published in 1956^3^, on the discovery of enterotoxigenic *E. coli*^4^.

De’s interest in cholera research and epidemiology took him to study patient care, epidemiology, infection, physiology, pathology, microbiology, and of course animal models. He studied the emergence of the El Tor biotype, phage resistance, haemolytic variants, haemagglutination, the anomalous Vogues-Proskauer reaction, and strains with unexpected surface features^1^. Today we might say he integrated the perspectives of host, pathogen, and the environment to encompass research areas that we still struggle to combine. In the academic world far from the disease, we have found it hard to direct research to meet the realities faced by the patient. Specialization has turned each subtopic into a discipline in its own right. Some areas proceed at a rapid pace while some stagnate, and we have had trouble applying innovation and resources to the next barrier in patient care. In the field of cholera, the crucial stumbling block was the erroneous dogma of systemic toxinosis.

## Stumbling from 1884-1959

Cholera was a backwater of research in 1959. Over the previous century, science had made little progress beyond an understanding of epidemiology, the aetiology of disease, and the empirical development of intravenous rehydration on a very limited scale. The inordinate delay in identifying cholera toxin allowed other bacterial pathogens to lead the way in basic scientific understanding and the development of vaccines and therapeutics. It had taken four years from the proposition of diphtheria toxin by Loeffler in 1884^5^, to its demonstration by Roux and Yersin in 1888^6^. For tetanus toxin, it took just five years from Nicolaier’s proposition in 1885, to Faber’s demonstration in 1890. For cholera, fully 75 years passed from Robert Koch’s proposal of a cholera poison or toxin in 1884, until the demonstration of enterotoxigenicity by De in 1959^6^–^9^. The delay was caused by many misconceptions and assumptions. For example, De noted: “Virchow (1879) described wholesale denudation of epithelium from intestinal villi in autopsies in cases of cholera.”^7^ In 1884 and 1885 Robert Koch maintained during the 1^st^ and 2^nd^ Berlin conferences on cholera that the disease was due to systemic multi-organ action of a toxin, and that cholera had a predominantly cardiovascular pathology. On the centenary of the Berlin conferences, van Heyningen described this as “Koch’s Blunder,” and a “serious matter”^8^,^9^. Koch recognized that stool was infective, but he never replicated the disease symptoms of diarrhoea and systemic dehydration in an animal despite a major effort, and he did not see it as important^8^. But it was important.

Koch’s authority undoubtedly inclined the first cholera vaccine developers, Jaime Ferrán y Clua (in 1885)^11^,^12^ and Waldemar Haffkine (from 1892)^13^ to exclusively use the parenteral route of administration, clearly compromising the safety, efficacy, and implementation of vaccination. Single-minded pursuit of intravenous fluid replenishment also limited the availability of treatment, and unnecessarily endangered patients with unbalanced and non-sterile infusions. Researchers made no progress in modeling cholera in animals with parenteral injections. By the 1930s, the received wisdom invoked the action of an endotoxin in cholera, and not an exotoxin. In the 1950s the mucinase hypothesis was in fashion. Nobelist Lord Florey of penicillin fame said to van Heyningen^8^: “The cholera problem is quite simple - the cholera mucinase stripped the protective layer of mucin from the intestinal epithelium, so cholera should be looked at as a kind of internal third degree burn, and no wonder that all that fluid poured into the gut”.

The whole field was in such confusion that van Heyningen and Seal give up in despair: “…therefore every conceivable preparation young, old, heated, dried, chemically treated cultures, their filtrates, and their bacterial cells, or extracts of them, was injected by every conceivable parenteral route, intravenous, intraperitoneal, subcutaneous, intramuscular, and more – into an astonishing array of animals…. They make dreadfully confusing reading and it would be carrying a sense of duty to the point of obsession to cover all the ground again, since all this effort was to little avail.” By little avail, they meant completely useless^8^. This was one that many got wrong^9^,^14^, and as a consequence, science could offer nothing to mitigate the three cholera pandemics that afflicted the period from 1884-1964.

## De revolutionising understanding of cholera pathogenesis

Finally, De independently re-invented the rabbit ileal loop assay^15^, combined it with cholera preparations, and proved the existence of cholera exotoxin^7^. In confirming De’s work, Carpenter concluded: “The pathogenesis of cholera is basically simple. The cholera victim ingests viable *V. cholerae*. The organisms multiply in the small bowel and produce an exotoxin which acts upon the mucosal cells of the small bowel, causing them to secrete large quantities of isotonic fluid. The small bowel produces isotonic fluid faster than colon can absorb it, and the result is a watery isotonic diarrhea. The rapid gastrointestinal loss of isotonic fluid is responsible for all the clinical manifestations of the disease”^16^.

The discovery was not so much ahead of its time as desperately awaited, but still De’s new model of pathogenesis had surprisingly little immediate impact. After a lag period of several years it was accepted, and a fresh generation of investigators boosted cholera from scientific obscurity to paradigm status. The development of therapy was also delayed, even though the discovery of cholera toxin and sodium/glucose co-transport firmly established the scientific rationale behind the tolerance and effectiveness of oral rehydration^17^. It took until 1973 for the misconceptions in vaccination to be recognized^18^ and undone by the World Health Organization when it withdrew its requirement for parenteral cholera vaccination, and it was 1974 before comparative studies were conducted that eventually led to the licensure of the first safe, effective, and orally-administered cholera vaccine^19^.

## Delayed recognition

It remains a mystery why De’s paper was hardly noticed at first. Incredibly, there were only 3 citations between 1959 and 1963. One citation from 1959 reported that De’s result could not be replicated, and proceeded to present a complicated model of toxinosis based on endotoxin, mucinase, and acetic acid to add to the bewildering range of contradictory studies already in the literature^20^. De’s work was cited by the researchers who subsequently purified and characterized cholera toxin, but the lack of professional recognition conspired with an inherent medical conservatism to leave patient care mired in the past. Carpenter held conservative elements responsible for irrational adherence to dogma and tradition in delaying the adoption of oral rehydration therapy, and accused them of “benign homicide”^17^. Lederberg agreed: “Our appreciation for De must then extend beyond the humanitarian consequences of his discovery. It is appalling to consider the millions of needless deaths that stem from the reign of “Toxins Kill”; no less than those that flow from the still imperfect application of means of rehydration. But he is also an exemplar and inspiration for a boldness of challenge to the established wisdom, a style of thought that should be more aggressively taught by example as well as precept”^14^. As an example of the most parsimonious explanation, van Heyningen and Seal said of De’s 1959 paper^7^: “This short essay deserves to go down as a classic in the history of cholera, and, as later developments have shown, in the history of cellular physiology and biochemistry.“ And directly to Professor De, van Heyningen wrote^10^: “I think your work was of tremendous significance and most serious researchers on cholera throughout the world acknowledge this….”.

Garfield^21^ reflected on the significance of S.N. De’s papers, two of which eventually attained “citation classic” status. Garfield proposed the concept of delayed recognition, and concluded that De’s 1959 paper in *Nature*: “while initially unrecognized, today is considered a milestone in the history of cholera research.” And he made a worrying suggestion that excellent science published by developing country scientists is often overlooked in favour of later work published by scientists from developed countries. But many investigators struggle for the recognition of research groups working just a few doors away, let alone those in far away lands of which they have no knowledge. It may be equally true that developing country scientists are also prone to overlook their colleagues’ work.

There are other possibilities. Finkelstein quoted the physicist Max Planck^22^: “An important scientific innovation rarely makes its way by gradually winning over and converting its opponents: it rarely happens that Saul becomes Paul. What does happen is that its opponents gradually die out and that the growing generation is familiarized with the idea from the beginning.” And so when cholera exotoxin finally was taken for granted, research leapt forwards, and S. N. De’s pivotal role fell into obscurity. De did express disappointment at the lack of recognition by his colleagues, and told van Heyningen that he was frustrated by a lack of support, facilities and staff to purify the toxin, obtain toxin from El Tor strains, and stabilize his strain collection. He wrote: “workers in developed countries cannot imagine how difficult it is to carry out and continue research work without willing personnel and without equipment in an undergraduate teaching department in a country like ours”^23^. But that is of course why the terms “resource-poor” and “cholera” go together. With minimal institutional investment, De’s discovery could have been solidified and credit properly accorded six or seven years earlier, with major consequences. Nonetheless, the essential literature from the period confers on S. N. De the credit for discovering cholera enterotoxin with the rabbit ileal loop.

The explanation for delayed recognition in this case may be much simpler. According to van Heyningen and Seal, it took the direct intervention of the U. S. National Institutes of Health to find out the merits of the cholera exotoxin theory. NIH approached Jack Craig in April 1963 to evaluate the evidence for cholera toxin. Craig was cognisant of toxins having studied the Iota toxin of *Clostridium welchii* in London. His investigations took him to S.N. De, and in October 1965 Craig reported what turned out to be cholera toxin activity to the U.S.-Japan Cholera Conference in Honolulu. Dr James A. Shannon, the Director of the NIH, understood the significance of the question to NIH-funded efforts toward developing cholera treatment in Dhaka and Kolkata, and he ordered the Cholera Advisory Committee to evaluate the exotoxin theory^8^. Jack Craig said: “No matter how simple it may now seem, we are compelled to recognize that this was a truly creative and novel piece of work, which started a chain of events which, in turn, forever altered our concepts surrounding the pathogenesis of secretory diarrhea”^24^.

## The narrowing of scholarship

Unfortunately situations like this could occur again, frustrating efforts to translate research findings into clinical interventions. According to James Evans^25^, scientists now tend to cite fewer journals and more recent articles, and more of those citations are to fewer journals and fewer articles. Evans suggested that scientists search the literature through hyperlinks rather than browse paper journals, arguing that searching online strengthens the prevailing opinion, accelerates consensus and narrows the range of findings that ideas are built upon. This is exactly the ‘Group-Think’ that Lederberg despised. And with new papers on cholera appearing daily, the field risks losing its long-term memory: narrowing, and specializing at an ever-greater pace.

Evans was naturally contradicted and the completely opposite hypothesis proposed by Gringras *et al*^26^, who found that citations were becoming deeper and broader. In any event, a Royal Society report^27^ showed British academics at least, to loathe any citation analysis because it is: “flawed to the point of being both misleading and inherently absurd”. Scientists like peer review and “indicators of esteem”. But esteem is not easy to measure without the wisdom of passing time. Honours are fraught with risks and there have been serious errors at the highest levels. For positive indicators of esteem, S. N. De’s friend and mentor Roy Cameron had them in abundance: a knighthood, Fellow of the Royal Society, Head of Department in University College Hospital, The Royal Medal, and a bust of himself at the Royal College of Pathologists that he led as founding president. S. N. De earned a D.Sc., was invited to a 1978 Nobel Symposium, was apparently nominated for the Nobel Prize in Physiology or Medicine more than once, had his admiring friends, and now has his own bust and a conference in tribute. But the recognition of his peers at the time was lacking. Is the lesson to embark on a campaign of self-publicity? Probably not. Cameron disdained his awards, finding satisfaction in fulfilling his duty. The famously introverted Barbara McClintock worked in isolation, was elected to the National Academy of Sciences and became a Nobel Laureate in 1983^21^. S.N. De also personified the fact that the stunning power of science needs no bluster. The true measure of esteem is how a discovery transforms strategic scientific thinking, and this can take time.

## Lack of recognition and the league of extraordinary ladies and gentlemen

Lack of recognition in cholera is not confined to scientists from developing nations. There are American examples of important but unappreciated applications of scientific discovery. According to van Heyningen and Seal, in 1883 when Kendrick’s famous cartoon was published depicting the spectre of cholera looming from Europe over New York, medical science in the US was not asleep, it was non-existent. The 5^th^ cholera pandemic raged, and Europe was in the Golden Age of Microbiology^8^.

Joseph Kinyoun graduated from New York University in 1882, joined the Marine-Hospital Service in 1886, and was posted to New York. With concern in America about importation of cholera, young Kinyoun traveled to Europe to study with Elie Metchnikoff and Robert Koch. Returning in 1887, he set up a one-room bacteriological “hygenic laboratory” within The Marine-Hospital on Staten Island, NY based on the facilities he had seen in Germany. Kinyoun was appointed laboratory director in 1887 in time for a flood of cholera cases among thousands of immigrants arriving onboard almost every ship from Europe^28^. Kinyoun used the techniques he had learned in Europe, and became the first American to isolate *Vibrio cholerae*, using his Zeiss microscope to demonstrate vibrios to his colleagues as confirmation of their clinical diagnoses^29^. In 1894, Kinyoun returned to Europe to learn the techniques of antitoxin therapy for diphtheria in Emile Roux‘s laboratory in *l‘Institut Pasteur*. He gained a Ph.D. from Georgetown University in Washington D.C. and successfully disseminated diphtheria treatment regimens across the USA. Kinyoun was then sent to the quarantine station on Angel Island in San Francisco Bay by the U.S. Surgeon General, where he identified a plague outbreak among the city‘s Chinese population. When he tried to institute controls, the California Governor blocked his efforts, denied the existence of the outbreak, and blamed Kinyoun. Kinyoun was transferred to serve in Detroit, Yokahama and Hong Kong, before resigning in 1903^30^,^31^. A parting address acknowledged Kinyoun thus: “…few men have done so much for the advance of Medical Science…[as] this modest, unassuming scientist…” whose: “…first and most important duty was loyalty to his country…”^32^.

While this was going on, Congress appropriated funds to expand the research mission of the Marine-Hospital Service. Joseph J. Kinyoun therefore has the best claim on being the first Director of what was to become the National Institutes of Health^30^,^31^. It is tempting to speculate that the American commitment to biomedical research began with Kinyoun’s little known isolation of *V. cholerae* and his success in preventing cholera from entering New York City in 1887.

Today, the sprawling campus of the NIH and the U.S. National Naval Medical Center face each other in Bethesda. The anchor of a coast guard cutter at the entrance to the NIH campus symbolizes the origins of the agency in the Marine-Hospital Service and its efforts to keep the 5^th^ cholera pandemic out of New York City. The present Director Francis Collins manages an annual budget of over $30 billion from his office in the James A. Shannon building, named after the NIH Director who saw the significance of exotoxin and made the Cholera Advisory Committee thoroughly evaluate the theory first proposed by S.N. De^8^. Joseph Kinyoun, a leader of the nascent American national biomedical effort, and the first American to isolate V. cholerae is remembered with a lecture series. Shannon is remembered with a building, but their lasting strategic impact on unrelenting cholera is not widely known or celebrated. Ultimately our esteem is expressed in our determination to attack the next hurdle standing in the way of our over-riding strategic objectives. Actions focused on effective public health solutions, such as those so presciently described by Nair and Takeda^32^, will be those most warmly recalled by history.

## Conclusion

In conclusion, a De in the life of cholera was very bad for cholera, very good for humanity. Paying tribute to De finally accords him the credit he deserves and corrects a long-standing unfairness. It also helps us recognize past mistakes, understand how to challenge dogmatic thinking, and not make reaching our next goals any harder than they already are. The career of S. N. De illustrated issues in science and public health that persist today. It remains important for programmes to listen as carefully to the quiet voices as to the loud ones, to develop and reward outstanding mentoring, training, and collaboration, to use simple, integrated strategies to meet long-term institutional development needs, long term concepts, short term projects, and special opportunities. We need to emphasise communications more than ever, we need to balance the value of senior leaders with the needs of young scientists to be independent as soon as they are ready, and we must share resources to study disease in a thoroughly integrated way. These are needs that will not go away and they require effort.

At the end his presentation to the Nobel Symposium to which he was invited in 1978, S.N. De said: “Chairman and Friends, before I conclude I wish to make a few personal remarks. I have been dead since the early 1960s, I have been exhumed by the Nobel Symposium Committee and these two days with you make me feel that I am coming to life again”^33^. If we do some or all of the things above, many more will have good reason to join us in our sincere tribute and thanks to Kolkata’s Dr S.N. De.
